# Antenna Placement Optimization for Distributed MIMO Radar Based on a Reinforcement Learning Algorithm

**DOI:** 10.1038/s41598-023-43076-z

**Published:** 2023-10-15

**Authors:** Jin Zhu, Wenxu Liu, Xiangrong Zhang, Feifei Lyu, Zhengqiang Guo

**Affiliations:** 1 School of Artificial Intelligence, Xidian University, Xi’an, 710071 China; 2 The 54th Research Institute of China Electronics Technology Group Corporation, CETC Key Laboratory of Aerospace Information Applications, Shijiazhuang, 050081 China

**Keywords:** Engineering, Electrical and electronic engineering

## Abstract

This paper studies an optimization problem of antenna placement for multiple heading angles of the target in a distributed multiple-input multiple-output (MIMO) radar system. An improved method to calculate the system’s coverage area in light of the changing target heading is presented. The antenna placement optimization problem is mathematically modelled as a sequential decision problem for compatibility with reinforcement learning solutions. A reinforcement learning agent is established, which uses the long short-term memory (LSTM)-based proximal policy optimization (PPO) method as the core algorithm to solve the antenna placement problem. Finally, the experimental findings demonstrate that the method can enhance the coverage area of antenna placement and thus has reference value for providing new ideas for the antenna placement optimization of distributed MIMO radar.

## Introduction

A distributed multiple-input multiple-output (MIMO) radar architecture is capable of cooperative work and signal processing, giving the system a strong ability to detect targets in a variety of environments^1–3^. Nevertheless, the issue of system resource configuration is crucial since sensible deployment of the antennas is necessary to maximize the system detection capabilities. Therefore, the optimal positioning of antennas must be studied to enhance radar performance^4–20^. The antenna placement issue can be solved in two steps. The first is to establish a standard for measuring the effectiveness of a radar system. On the basis of this standard, an optimization problem will be formulated and solved using a suitable technique.

### Related works

In related works, researchers have studied antenna placement optimization problems based on various criteria, including localization accuracy^4–6^, system output signal-to-noise ratio (SNR)^7,8^, target tracking performance^9,10^, coverage area of the surveillance zone^11–16^, and combinations of any two of these performance metrics^17,18,20^. Among the above criteria, the performance metric of the system output SNR cannot be used to evaluate a situation in which the target distance varies within a certain range. Furthermore, only the placement of transmit antennas has been investigated^7,8^. However, in distributed MIMO radar systems, it is necessary to account for the positioning of both the transmit and receive antennas. Target detection is the foundation for target location, tracking and other applications. The extent of the coverage area can be used to characterize a system’s target detection capabilities. Therefore, to site the antennas optimally, the coverage area is considered as the criterion. X. Sun et al. used a coverage ratio as the objective function to build an optimization problem and evaluate the surveillance performance^11^. Li et al. constructed a dynamic multi-objective optimization problem for antenna deployment by choosing the effective coverage rates of different non-fixed surveillance regions as objective functions^12^. A series of studies have been carried out by Y. Wang et al. in which a coverage ratio metric is used as the optimization objective function to solve antenna placement problems with different constraints^13–16^. However, none of those studies considers the state of the target, and they all calculate the coverage ratio based on a single heading angle. This is a shortcoming because in a distributed MIMO radar system, the heading angle of the target may change, in turn causing the target radar cross section (RCS) to change, which should be considered when setting the optimization criterion.

There are also some problems with the optimization methods used in related research. In practice, distributed MIMO radar antennas are widely distributed in a certain area, and the surveillance area extends to hundreds of kilometres. The antenna placement problem essentially concerns a combination of antennas at different positions. A wider deployment area provides more possibilities for the antenna positions. Therefore, the optimization of antenna placement is a highly complex combinatorial optimization problem and cannot be solved by deterministic methods such as a sequentially exhaustive enumeration (SEE) method^19^ because of the high computational load. Particle swarm optimization (PSO) has been used to solve the problem of antenna placement^13–16^. However, it has the disadvantages of poor search accuracy and local search ability. Genetic algorithms (GAs) are also a classical type of heuristic algorithms that have been used to solve antenna placement problems^11^. A GA has a great advantage in reducing the computational load for such a problem but can easily fall into a local optimum.

Compared to evolutionary algorithms, reinforcement learning is a different approach in which a sequential decision model is adopted to solve an optimization problem. When the space of possible policies is large, as in the problem addressed here, a reinforcement learning algorithm is more advantageous. For MIMO radar, the relative positioning between antennas will affect the interaction between them and ultimately affect the system coverage area. Methods that are able to exploit the details of individual behavioural interactions, such as reinforcement learning algorithms, can be much more efficient than evolutionary methods in many cases^21^ when the challenge to be solved is a complex high-dimensional combinatorial problem. A reinforcement learning scheme consists of three fundamental elements: state, action, and reward. An agent takes an action by comprehending its current state, thereby triggering a state transition, and thus learns to optimize the value of its actions. Finally, the agent learns to maximize the reward obtained for task completion. Reinforcement learning has been studied and achieved remarkable results in many fields, such as chess playing^22,23^, robot control^24–26^, and autonomous driving^27–29^. In this paper, the long short-term memory (LSTM)-based proximal policy optimization (PPO) method of reinforcement learning is introduced into MIMO radar antenna placement optimization and compared with a GA and a PSO algorithm.

### Our contributions and paper organization

In this paper, the antenna placement optimization problem in a distributed MIMO radar system is considered. The purpose of optimizing antenna placement is to maximize the coverage area of the radar system, thereby improving the radar search performance. First, a better way is presented to determine the system’s coverage area by accounting for the target’s changing heading angle. This approach yields an objective function to be used in solving the antenna positioning problem. Then, the LSTM-based PPO method of reinforcement learning is introduced to address the challenge of the highly nonconvex and combinatorial nature of the problem. Modelling ideas are discussed, and a reinforcement learning agent is established. Finally, numerical results show that the proposed method achieves superior surveillance performance compared to a GA and a PSO algorithm.

## Model of system detection performance

### Scene description

A coverage area metric is usually considered as the criterion to evaluate the surveillance performance of a radar system. In related works^11–16^, the coverage capability of such a system has been calculated by assuming the target to travel at a single heading angle. In practice, however, the target heading is unknown and dynamic. Hence, each possible heading angle of the target has equal probability in the system. However, the detected target is usually moving towards the radar station within a certain range of angles. As a result, all angles in this angle range are possible heading angles and can be considered to have equal probability. Therefore, an angle range is assumed, and multiple heading angles in this angle range will be considered to calculate the coverage area and used to optimize the performance of the radar system. It’s assumed that there are several targets in the surveillance area of the radar system. When they can be distinguished by the system, the number of targets does not affect the coverage area because their echo will be processed separately. If they cannot be distinguished from each other, the radar system will treat them as one target and the problem of distinguishing among multiple targets is out of the range of the antenna placement optimization based on the coverage area. Therefore, a single target will be used as an example for the study of radar system coverage area optimization in this paper.

**Figure 1:**
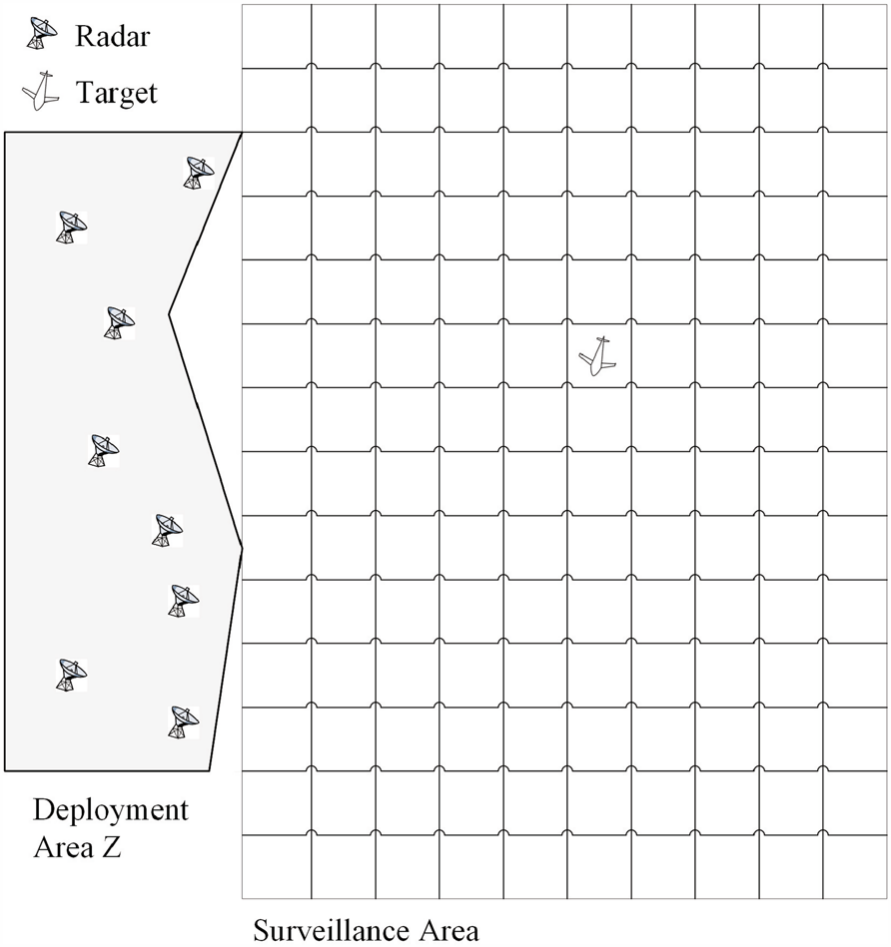
Sketch of the surveillance area and deployment area.

It is assumed that there are *N* radar antennas distributed in an area Z in the 2D plane. Considering the regional limitations of radar system deployment in practical applications, area Z is set to an irregular polygon. This assumption is similar to an actual radar placement area because there are usually terrain and altitude limitations preventing the establishment of a regular region in which to place antennas. However, this design will increase the optimization difficulty. On the right side of Z is the surveillance area of the system. The surveillance area is divided based on radar range cells. Hence, the surveillance area is actually a set of grid cells. A sketch of the surveillance area and deployment area is given in Fig. 1.

The deployment area Z is assumed to be divided into *U* grid cells and formed the matrix $\textbf{Z}_U$. The surveillance area is divided into *L* grid cells and formed the matrix $\textbf{S}_L$. The target can rotate through *M* angles and the angle matrix is defined as $\textbf{A}_M$. The *i*-th transmit antenna is at ${(x_i,y_i)}$ and the *j*-th receive antenna is at ${(x_j,y_j)}$. The coordinates of the *l*-th grid cell are ${(x_l,y_l)}$. The coordinates of above antennas and grid cell satisfy

1 $$\left\{ {\begin{array}{*{20}l} {\left( {x_{i} ,y_{i} } \right) \in {\mathbf{Z}}_{U} ,1 \le i \le N,} \hfill \\ {\left( {x_{j} ,y_{j} } \right) \in {\mathbf{Z}}_{U} ,1 \le j \le N,} \hfill \\ {\left( {x_{l} ,y_{l} } \right) \in {\mathbf{S}}_{L} ,1 \le l \le L,} \hfill \\ \end{array} } \right.$$

respectively. Then, the distance ${R_{i,l}}$ between the *i*-th transmit antenna and the *l*-th grid cell and the distance ${R_{j,l}}$ between the *j*-th receive antenna and the *l*-th grid cell can be calculated by

2 $$\left\{ {\begin{array}{*{20}l} {R_{{i,l}} = \sqrt {(x_{i} - x_{l} )^{2} + (y_{i} - y_{l} )^{2} } ,} \hfill \\ {R_{{j,l}} = \sqrt {(x_{j} - x_{l} )^{2} + (y_{j} - y_{l} )^{2} } ,} \hfill \\ \end{array} } \right.$$

respectively.

The RCS data of the target is given in the form of a two-dimensional matrix ${\Delta }$ which satisfies

3 $$\Delta = \left\{ {\delta _{{\theta ,\phi }} \in \Delta |0 \le \theta \le 2\pi ,0 \le \phi \le 2\pi } \right\}.$$

$\theta$ means the incidence angle and $\phi$ means the reflection angle. When the target is in the *l*-th grid cell and oriented at the *m*-th angle $\alpha _m$, which satisfies $\alpha _m\in \textbf{A}_M, 1\le {m}\le {M}$, every transmit-receive (TR) channel forms different incident angles and reflection angles. The incident angle $\theta _{i,l,m}$ formed by the *i*-th transmit antenna and the reflection angle $\phi _{j,l,m}$ received by the *j*-th receive antenna at the *l*-th grid cell with rotation angle $\alpha _m$ can be obtained by

4 $$\left\{ {\begin{array}{*{20}l} {\theta _{{i,l,m}} = {\text{arctan}}\frac{{y_{i} - y_{l} }}{{x_{i} - x_{l} }} - \alpha _{m} ,} \hfill \\ {\phi _{{j,l,m}} = {\text{arctan}}\frac{{y_{j} - y_{l} }}{{x_{j} - x_{l} }} - \alpha _{m} ,} \hfill \\ \end{array} } \right.$$

respectively. The RCS of target at the *l*-th grid cell with the rotation angle $\alpha _m$ for the $i-j$ TR channel can be obtained as $\delta _{\theta _{i,l,m},\phi _{j,l,m}}$. As shown in Eq. (4), different TR channels obtain different RCS values of the target. According to the bistatic radar equation, the SNR of the $i-j$ TR channel when the target is in *l*-th grid cell with rotation angle $\alpha _m$ can be expressed as^13^

5 $$\xi _{l}^{{i,j,m}} = \frac{{P_{t} G_{t} G_{r} \lambda ^{2} \delta _{{\theta _{{i,l,m}} ,\phi _{{j,l,m}} }} }}{{\left( {4\pi } \right)^{3} kT_{o} B_{n} R_{{j,l}}^{2} R_{{i,l}}^{2} }},$$

where ${{P}_{t}}$ is the transmit power of a radar antenna; ${{G}_{t}}$ and ${{G}_{r}}$ denote the gains of the transmit and receive antennas, respectively; $\lambda$ represents the signal wavelength; *k* is the Boltzmann constant; ${{T}_{0}}$ is standard room temperature, generally 290 K; ${{B}_{n}}$ is the noise bandwidth.

When the target is in the *l*-th grid cell, the SNRs of all the ${{N}^{2}}$ TR channels can be calculated following Eq. (5). The ratio of all the echo energy to noise power, $\xi _l$, is calculated as the sum of the echo SNRs ${{\xi }_{l}^{i,j,m}}$ of all channels at all angles^30,]^^31^, which is given by

6 $$\xi _{l} = \sum\limits_{{i = 1}}^{N} {\sum\limits_{{j = 1}}^{N} {\sum\limits_{{m = 1}}^{M} {\xi _{l}^{{i,j,m}} } } } .$$

When the target is in the *l*-th grid cell, the detection probability of the radar system is expressed as^30,]^^31^

7 $$\left\{ {\begin{array}{*{20}l} {\overline{{\text{P}}} _{{\text{d}}} = Q_{{J \times J}} \left( {\sqrt {2 \times \xi _{l} } ,\sqrt {2 \times \gamma _{T} } } \right),} \hfill \\ {\overline{{\text{P}}} _{{{\text{fa}}}} = e^{{ - \gamma _{T} }} \sum\limits_{{n = 0}}^{{N \times N - 1}} {\frac{{\gamma _{T}^{n} }}{{n!}}} ,} \hfill \\ \end{array} } \right.$$

where *Q* means Marcum *Q*-function; $\gamma _T$ represents the detection threshold; $\bar{\text {P}}_\text {fa}$ represents false alarm probability. $\xi _l$ can be calculated by Eq. (6) and substituted into Eq. (7) to calculate the detection probability. If the calculated detection probability exceeds the set one, then the *l*-th grid cell is included in the detection range. By calculating the $\xi _l (l=1,2,3,...,L)$ for all grid cells and the corresponding detection probability, the grid cells of which calculated detection probability exceeds the set value will be obtained. The number of grid cells that meet the requirements is defined as the coverage area of the radar system.

### Mathematical model of the optimization problem

According to the method of calculating the coverage area, a mathematical model of the optimization problem can be established. The antenna placement area Z and the surveillance area are both divided into grids to form the discrete matrices $\textbf{Z}_U$ and $\textbf{S}_L$, respectively, with *U* grid cells in antenna placement area Z and *L* grid cells in the surveillance area. Note that when the target, area Z and the surveillance area are determined, a change in the placement of the radar antennas will influence ${R_{i,l}}$, ${R_{j,l}}$ and ${\delta _{\theta _{i,l,m},\phi _{j,l,m}}}$, further affecting the echo signal power and coverage area. Therefore, the antenna positions need to be optimized to maximize the coverage area. The corresponding optimization problem can be expressed as follows:

8 $$\mathop {\max }\limits_{{\mathbf{P}}} {\mkern 1mu} {\text{ }}C\left( {\mathbf{P}} \right){\text{ }}s{\text{.}}t{\text{. }}p_{n} \in {\mathbf{Z}}_{U} ,{\text{ }}1 \le n \le N,$$

where $\textbf{P} = [{p_1},{p_2},...,{p_N}]$, ${p_n} = ({x_n},{y_n})$ denotes the coordinates of the n-th antenna, and $C\left( \textbf{P} \right)$ represents the coverage area calculated under antenna placement $\textbf{P}$. A larger $C\left( \textbf{P} \right)$ indicates better surveillance performance under antenna placement $\textbf{P}$. Accordingly, an algorithm for optimizing the antenna placement to maximize the coverage area will be studied in the next section.

## Antenna placement optimization based on the LSTM-based PPO method

### Modelling ideas

Based on the performance model previously described, the objective of the antenna placement optimization problem is to determine a cluster of antennas in a given deployment area whose distribution maximizes the coverage area in the surveillance zone. This is obviously a combinatorial optimization problem with a relatively large solution space. In this paper, the problem is transformed from a combinatorial optimization problem into a sequential decision problem, making it compatible with reinforcement learning solutions.

The three elements of reinforcement learning are specified as state, action, and reward. The state represents the coverage area corresponding to the current antenna distribution, the action is the position of the newly added antenna, and the reward is proportional to the added coverage area (the state, action, and reward will be explained in detail later). The modelling process is shown in Table 1. By applying this process of adding antennas one-by-one in accordance with the state, we model the problem as a sequential decision problem, laying the foundation for reinforcement learning algorithms.

**Table 1:** The modeling process of the LSTM-based PPO algorithm.

Modeling process
$$\textbf{for}$$ iteration = 1, 2, ..., *N* $$\textbf{do}$$
i. Decide where to add an antenna based on the current state
ii. Take the corresponding action (add an antenna)
iii. Update the state (the coverage area)

### Reinforcement learning agent for antenna placement optimization

In this paper, reinforcement learning methods are used to solve the antenna placement problem. A reinforcement learning agent that decides where to place antennas is finally developed through iterative training. The core algorithm of this agent is the LSTM-based PPO method.

The PPO algorithm is a novel deep reinforcement learning algorithm proposed by Schulman^27^, which combines a policy gradient algorithm with an actor-critic (AC) architecture. It can be applied in continuous state and action spaces^25^. It is used as the default reinforcement learning algorithm of OpenAI due to its stability, ease of training and outstanding performance in many tasks.

An LSTM network is a special type of recurrent neural network that can learn about long-term dependencies. The LSTM architecture was proposed by Hochreiter and Schmidhuber and has been improved and developed by numerous researchers in a series of subsequent works^27^. It is now widely used and works incredibly well for many timing-related issues^28^. The reason why LSTM models can solve problems with long-term dependencies is the introduction of a gate mechanism for controlling the data flow. The LSTM architecture includes three kinds of gates, namely, forget, input, and output gates, corresponding to different operations. The forget gate determines what information needs to be discarded from the cell state. The input gate combines the hidden state and the current input to update the cell state. The output gate determines what the next hidden state will be.

The PPO algorithm usually uses a fully connected neural network for feature extraction. However, in a MIMO system, each radar antenna transmits a mutually orthogonal signal. Thus, each radar antenna can receive multiple echo signals simultaneously and separate the signals from itself and other antennas. The signals in each TR channel are independent. Accordingly, when antennas are deployed following the modelling process in Table 1, each newly placed antenna will form a new TR channel with the previously placed antennas and affect the current state, that is, the coverage area. As a result of these interactions, the prediction of the radar antenna positions is naturally characterized by long-term dependencies. Therefore, the LSTM-based PPO algorithm is used to solve this problem. The LSTM network is used as a feature extractor to extract features from states and output useful perceptual information. It can enhance the ability to learn the temporal features of the distributed MIMO radar coverage area^32^; then, on this basis, the policy function and value function can be approximated by a fully connected neural network.

### Agent

**Figure 2:**
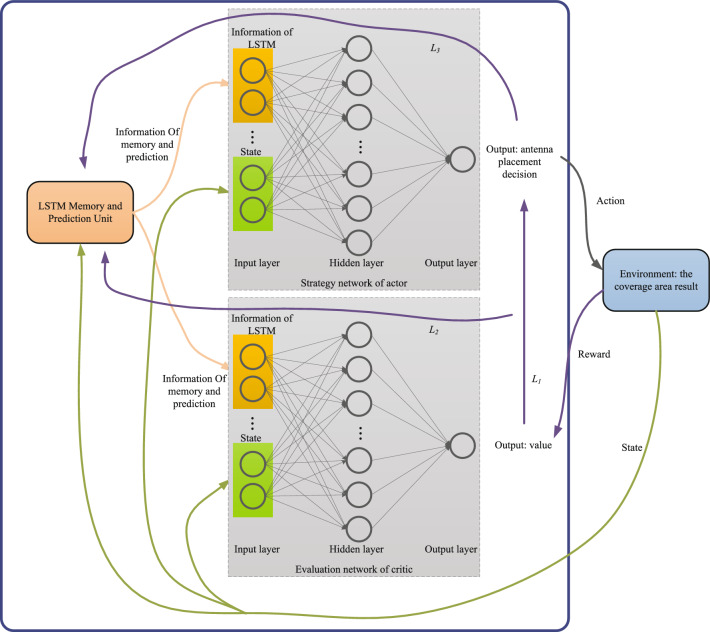
Composition and working process of the agent.

The agent interacts with the environment by executing actions and obtaining rewards to be trained for decision making. The agent in the LSTM-based PPO algorithm is an AC structure type agent as shown in Fig. 2. It consists of three modules, including LSTM memory and prediction unit, strategy network of actor, and evaluation network of critic. Actor makes the antenna placement decision according to the current coverage of the surveillance area, and critic evaluates the advantages and disadvantages of the placement decision. The strategy module uses a feedforward neural network to approximate the optimal scheduling strategy. The evaluation module also uses a feedforward neural network as a value network to approximate the real value function. The environment that interacts with the agent is the coverage area calculation function. In each round, the agent performs *N* times of one antenna placement. Based on the objective function, the agent interacts with the environment after each placement to obtain the current coverage area, and then obtains the corresponding reward and loss functions. Through multiple rounds of decision training, the agent will finally obtain the optimal antenna placement decision.

### State

The state is defined as the current radar coverage area. As mentioned in Sect. “Scene description”, the surveillance area is divided into *L* grid cells, and in each grid cell, there are *M* angles of the target to cover. For each grid cell, if the corresponding detection probability exceeds the set value, this grid cell is considered to be included in the detection range. If a grid cell is included in the detection range, its state is marked as 1; otherwise, the state is marked as 0. Thus, a two-dimensional matrix consisting of values of 0 and 1 is obtained. This two-dimensional matrix is then stretched into a one-dimensional vector because LSTM networks accept only one-dimensional vectors as input.

### Action

The action is defined as the next antenna placement location. As mentioned in the scene description, the deployment area is set to an irregular polygon. Actions are coded in the following way:

1. The deployment area is divided into two parts at the middle.
2. The deployment area is gridded for actions.
3. The range of [−1,1] is used to encode the antenna position, with values of [−1,0) corresponding to the left side of the deployment area and [0,1] corresponding to the right side.

### Reward

The reward is related to the previous state, the current state and the number of antennas to be placed, denoted by *N*. When *N* antennas are uniformly deployed on the right boundary of the radar deployment area, the corresponding coverage area, denoted by *d*, is calculated as a reference for the reward function. Suppose that the antenna coverage area in the previous state is *a* (the number of grid cells with state value 1), and the antenna coverage area in the current state is *b*.

When the first *N*/2 (or $(N + 1)/2$, if *N* is an odd number) antennas are being deployed, the reward function *r* is defined as follows:

9 $$\begin{aligned} r=b-a-{m}/{(2\times N)}. \end{aligned}$$

When the remaining antennas are being deployed, the reward function *r* is defined as follows:

10 $$\begin{aligned} r=b-a-2\times {m}/{N}. \end{aligned}$$

## Simulations and analysis

In this section, some simulations are presented to show the effectiveness of the proposed method.

### Simulation setup

**Figure 3:**
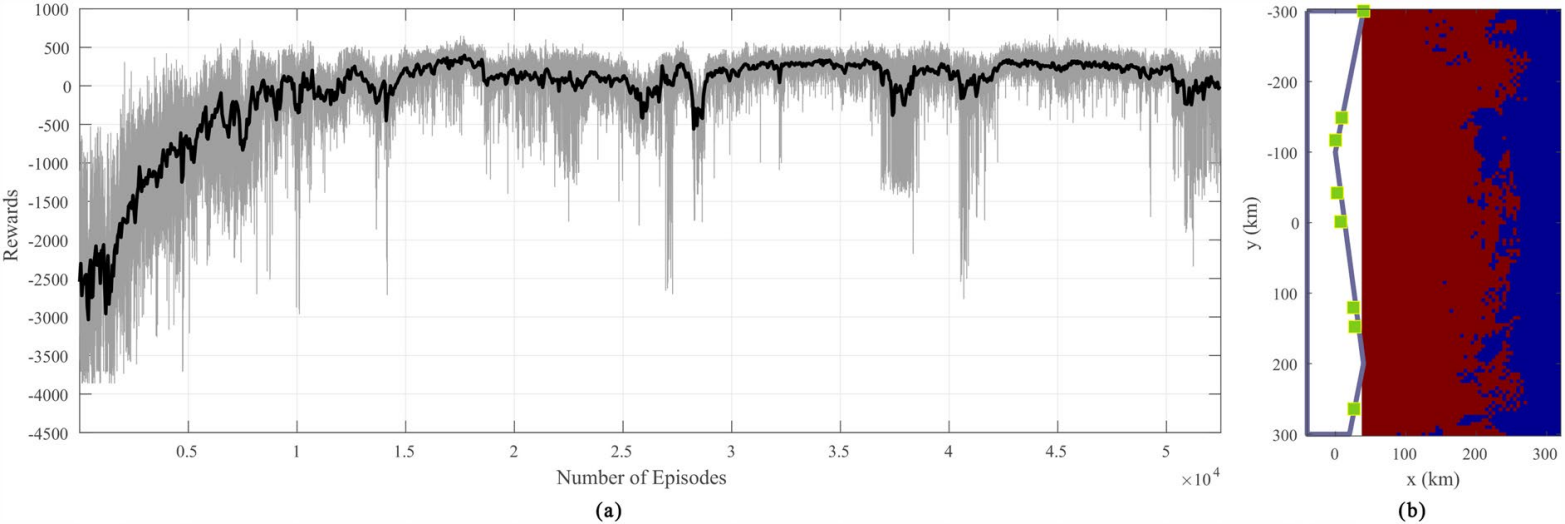
The reward curve, optimal antenna placement and coverage area obtain with the proposed method. (**a**) Reward curve, (**b**) Optimal antenna placement and coverage area.

The geographical limitations of antenna system deployment in practical applications should be accounted for. For this evaluation, the length of the radar antenna deployment area is set to 80 km, and the widest width of the irregular polygon is set to 40 km. The boundary case is the dull purple line shown in Fig. 3b, where the Cartesian coordinates of the irregular points are $({\text {20 km, 300 km}})$, $({\text {40 km, 200 km}})$, and $({\text {0 km, }}-{\text {100 km}})$. The length of the surveillance area is set to 250 km, and the width is set to 300 km. In the surveillance area, the target can rotate in the range of $- {15^ \circ }$ to ${15^ \circ }$, and the ${0^ \circ }$ direction of the target points along the negative direction of the y-axis. The total number of antennas deployed is $N = 8$. The detection probability ${P_d} = 0.8$ and the false alarm probability ${P_{fa}} = {10^{ - 6}}$ is used^33^.

**Table 2:** Parameter of the LSTM-based PPO algorithm.

Parameter	Value
Time steps	4.2$\times 10^{5}$
Learning rate	1$\times 10^{-3}$(multiplied by 0.3 when trained 1$\times 10^{5}$ and 2.6$\times 10^{5}$ steps)
LSTM hidden size	64
LSTM layers	2

The hyperparameters of the reinforcement learning algorithm are listed in Table 2. To verify the effectiveness of the algorithm, comparative experiments using a GA and a PSO algorithm are conducted. We set the population size for the GA to $S = 50$, the maximum number of iterations to ${T_{\max }} = 200$, the crossover probability to ${p_c} = 0.9$, and the mutation probability to ${p_m} = 0.1$^11^. In the PSO algorithm, the particle number, the inertia weight, the number of iteration, the individual learning factor and the group learning factor are set as 20, 1, 200, 2 and 2, respectively. Here, 20 GA and 20 PSO algorithm experiments are conducted.

What’s more, comparative experiments under different number of antennas are also conducted using the GA and the PSO algorithm. The effect of the number of antennas on coverage area and algorithm performance is analyzed.

### Results and analysis

The reward value of the LSTM-based PPO method over multiple training steps is shown in Fig. 3a. The reward value increases rapidly during pre-training and gradually during later stages, demonstrating how the algorithm learns the antenna placement logic. In the early stage, the algorithm does not have any knowledge and randomly deploys new antennas. During this period, the agent tends to deploy new radar antennas on the left side of the deployment zone; at this time, the reward value is extremely low. Throughout the process of training, the agent gradually begins to deploy antennas on the right side of the deployment zone and slowly approaches the right boundary of the deployment zone, achieving its goal of increasing the reward value. Based on the reward value, the optimal antenna placement is obtained under the proposed method, as shown in Fig. 3b.

In Fig. 3b, the dull purple line indicates the boundary of the radar antenna deployment area, and the green squares indicate the positions of antennas. The dark red area is the coverage area, and its value is 4663.

**Figure 4:**
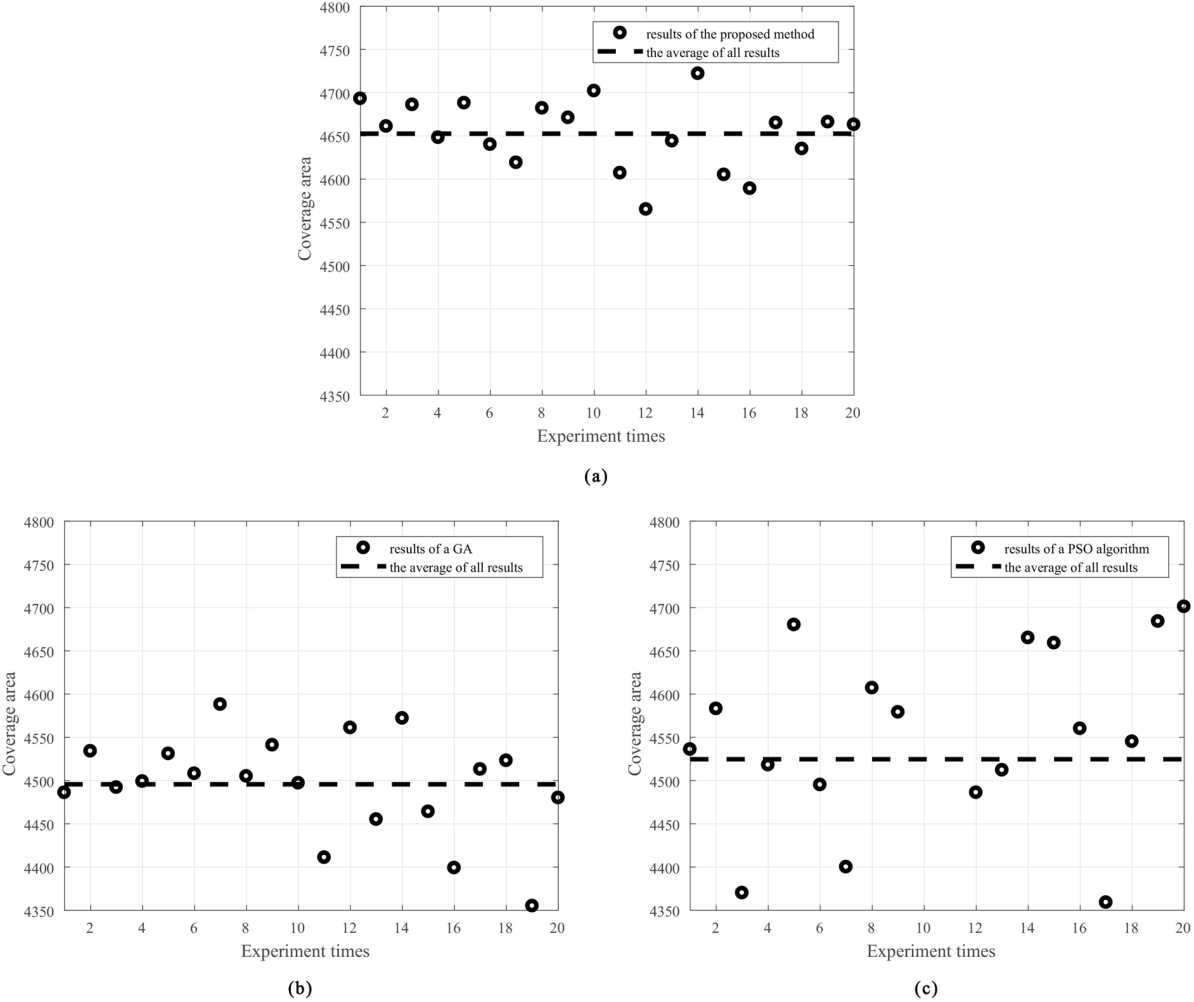
Experimental results of the proposed method, the GA and the PSO algorithm. (**a**) Experimental results of the proposed method, (**b**) Experimental results of the GA, (**c**) Experimental results of the PSO algorithm.

**Figure 5:**
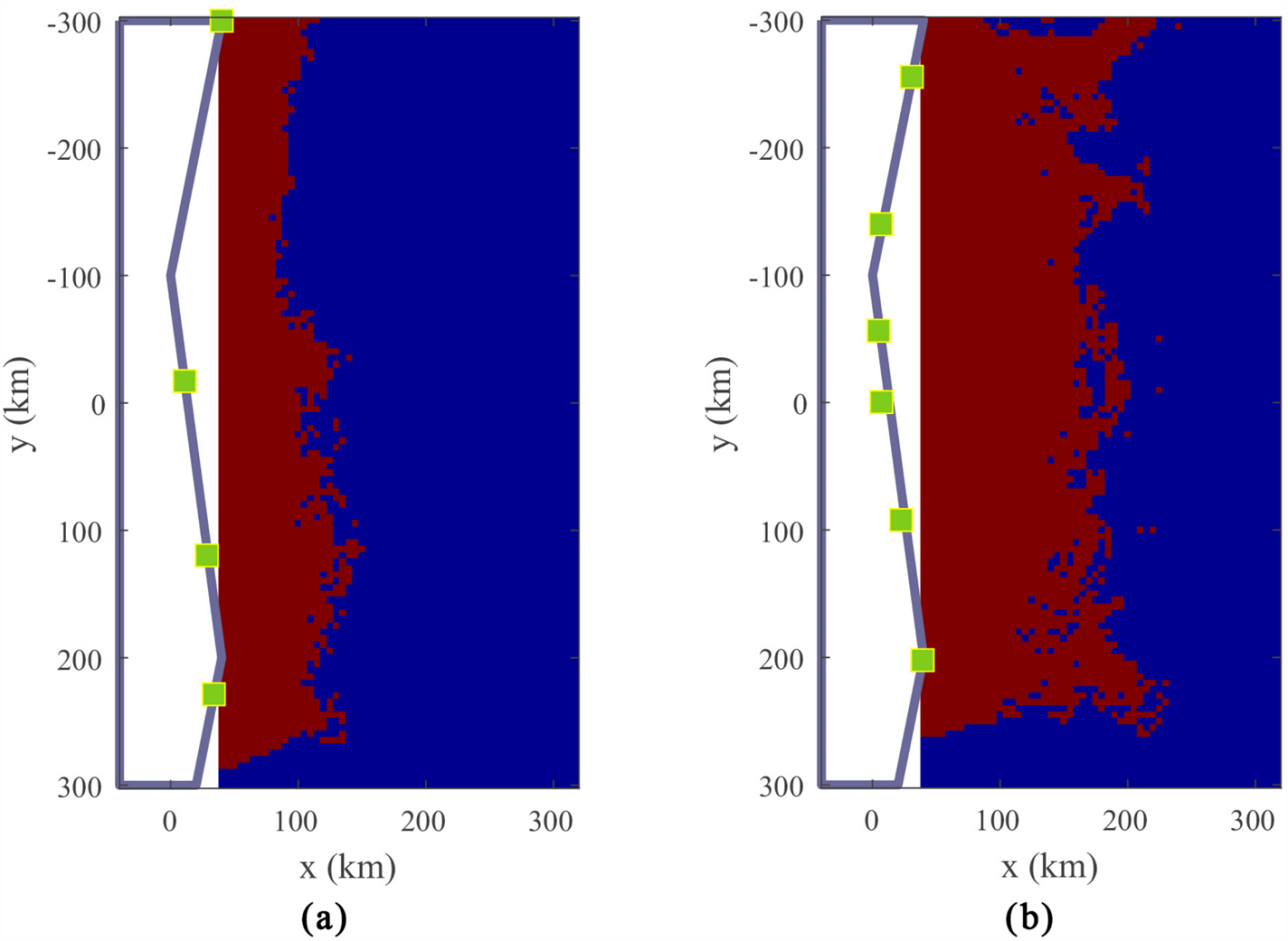
Experimental results of the proposed method with different number of antennas. (**a**) The coverage area results of 4 antennas, (**b**) The coverage area results of 6 antennas.

In total, 20 experiments are run using the proposed method, and 20 experiments are also run using the GA and the PSO algorithm for comparison. The results are shown in Fig. 4. In Fig. 4, the circles indicate the results of the individual experiments, and the dotted line represents the average of all results. The maximum values of coverage area achieved with the proposed method, the GA, and the PSO algorithm are 4722, 4588, and 4701, respectively. The average values of coverage area achieved with the proposed method, the GA, and the PSO algorithm is 4652.6, 4495.7, and 4524.6, respectively. The average area under the proposed method is 3.49$\%$ higher than that under the GA, and 2.83$\%$ higher than that under the PSO algorithm, which proves the superiority of the LSTM-based PPO method for solving the antenna placement optimization problem. According to the overall standard deviation formula, the standard deviation of the 20 experimental results of the proposed method is 40.22, corresponding to 0.86$\%$ of the average 4652.6; the standard deviation of the 20 experimental results of the GA is 58.04, corresponding to 1.29$\%$ of the average 4495.7; the standard deviation of the 20 experimental results of the PSO algorithm is 130.90, corresponding to 2.89$\%$ of the average 4524.6. These results show that the proposed method is also more robust than the GA and the PSO algorithm.

Experiments using the proposed method are performed by changing only the number of antennas while other parameters remain unchanged. The coverage area results of 4 and 6 antennas are shown in Fig. 5a,b, respectively. Comparing Figs. 3 and 5, it can be seen that the coverage area will increase with the number of antennas. Because more antennas mean more channel energy to accumulate, the sum of echo SNR increases and the detection probability increases.

To further verify the effect of the number of antennas on the proposed method, 20 experiments are conducted using the proposed algorithm, the GA and the PSO algorithm in the case of $N = 4$ and $N = 6$ antennas, respectively. The results are shown in Table 3.

Table 3 shows the average and the standard deviation of the optimal coverage area of the three algorithms in the case of different number of antennas. As the number of antennas increases, the coverage area also increases. The optimal coverage area obtained by the proposed method is larger than that obtained by the GA and the PSO algorithm. The advantage of the proposed algorithm is more obvious when the number of antennas is larger. This is because the higher the number of antennas, the higher the optimization dimension. The problem is also more complex. The proposed method is less likely to fall into local optimum than the GA and the PSO algorithm, and thus achieves better results.

**Table 3:** The optimal coverage area results of the three algorithm.

The number of antennas	The proposed method		The GA		The PSO algorithm	
	AVG.	S.D.	AVG.	S.D.	AVG.	S.D.
4	1648.4	0.69$\%$	1596.1	1.54$\%$	1608.3	2.20$\%$
6	3052.7	0.84$\%$	2890.2	2.12$\%$	2918.1	2.12$\%$
8	4652.6	0.86$\%$	4495.7	1.29$\%$	4524.6	2.89$\%$

### Analysis of computational complexity

For a reinforcement learning algorithm, the computational complexity is usually difficult to calculate. Moreover, during the coverage area optimization, the objective function, which includes the coverage area calculation function, is much more computationally intensive than the optimization algorithm itself due to multiple transceiver channels and large number of monitoring area grids. The proposed method for optimization is more computationally intensive because the reinforcement learning algorithm requires a lot of interaction with the environment to learn the antenna placement and thus obtain better coverage results. The process of one-by-one placement of the antennas requires more computations of the coverage area and is therefore more computationally intensive than the other two algorithms, which is a sacrifice to obtain a better coverage area.

## Conclusion

An optimization problem of radar antenna placement for multiple heading angles of the target was studied in this paper. An improved method of calculating the radar system’s coverage area was presented in light of the unknown target heading and the possibility of target manoeuvres at any time. Then, a mathematical model of the antenna placement optimization problem was established. A sequential decision model was constructed, and the LSTM-based PPO method of reinforcement learning was introduced to solve the optimization problem. According to the experimental results, this reinforcement learning method can deduce the inner logic of antenna placement and optimize the antenna placement scheme. The proposed method was compared with a GA and a PSO algorithm, and the experimental findings demonstrate that the proposed method can enhance the coverage area achieved through antenna placement and thus has reference value for providing new ideas for the antenna placement optimization of distributed MIMO radar systems.

## Data Availability

The data sets used and/or analyzed during the current study available are available in the https://figshare.com/articles/dataset/Datasets_for_the_paper/22584634.
